# Deep brain stimulation in Gilles de la Tourette syndrome: killing several birds with one stone?

**DOI:** 10.12688/f1000research.9521.1

**Published:** 2016-09-07

**Authors:** Andreas Hartmann

**Affiliations:** 1 Sorbonne Universités, UPMC Univ Paris 06, UMR S 1127, CNRS UMR 7225, Paris, France; 2 Assistance Publique-Hôpitaux de Paris, Department of Neurology, Groupe Hospitalier Pitié-Salpêtrière, Paris, France; 3 French Reference Centre for Gilles de la Tourette Syndrome, Groupe Hospitalier Pitié-Salpêtrière, Paris, France

**Keywords:** Tics, Tourette, deep brain stimulation (DBS), comorbidities

## Abstract

In patients with severe, treatment-refractory Gilles de la Tourette syndrome (GTS), deep brain stimulation (DBS) of various targets has been increasingly explored over the past 15 years. The multiplicity of surgical targets is intriguing and may be partly due to the complexity of GTS, specifically the various and frequent associated psychiatric comorbidities in this disorder. Thus, the target choice may not only be aimed at reducing tics but also comorbidities. While this approach is laudable, it also carries the risk to increase confounding factors in DBS trials and patient evaluation. Moreover, I question whether DBS should really be expected to alleviate multiple symptoms at a time. Rather, I argue that tic reduction should remain our primary objective in severe GTS patients and that this intervention may subsequently allow an improved psychotherapeutic and/or pharmacological treatment of comorbidities. Thus, I consider DBS in GTS not as a single solution for all our patients’ ailments but as a stepping stone to improved holistic care made possible by tic reduction.

Deep brain stimulation (DBS) has been used for over 15 years to treat severe forms of Gilles de la Tourette syndrome (GTS) refractory to pharmacological and, more recently, cognitive-behavioral therapies (CBT) ( Schrock *et al.*, 2015). Despite the relatively small numbers of patients operated so far, the number of surgical targets is impressive ( Porta *et al.*, 2013). The available double-blind trials favor the thalamus and the globus pallidus internus (both anteromedial and posteroventral parts) but the debate if these two are the best targets or if other targets need to be explored remains open ( Servello *et al.*, 2016). As we have learnt from Parkinson disease, establishing just one or two consensual DBS targets is a long endeavour which requires time and a large number of patients ( Lukins *et al.*, 2014). Providing the latter will certainly be difficult in a comparatively rare disease like GTS.

Why so many potential targets in GTS? One of the main reasons appears to be the wish to diminish not only tics but also comorbidities (obsessive-compulsive disorder (OCD), impulsivity, attention deficit hyperactivity disorder (ADHD), anxiety, depression and others) which are present in almost 90% of patients meeting DSM criteria for GTS ( Hirschtritt *et al.*, 2015). Specifically, these patients fall into the category named « full-blown GTS » by Robertson (2015) and are also the most likely candidates to undergo surgery. Thus, a tailor-made, individualized approach might indeed make sense instead of including/randomizing patients into studies where a certain diagnostic uniformity is required or at least assumed.

I will argue that in an admittedly complex situation, Occam’s razor is the way to go forward. First, there is no GTS without tics. Challenging DSM-5 criteria is understandable but unrealistic ( Robertson & Eapen, 2014). In clinical practice, however, even if DSM-5 criteria for GTS are met, we do of course establish the predominant symptoms in terms of impairment. Then, we chose the surgical target which we believe will be best suited to counter the main burden on the patient’s quality of life. This may mean that a patient with severe tics but even more severe OCD might actually be operated predominantly for the latter, targeting the subthalamic nucleus, for instance, which is not a usual target in GTS ( Mallet *et al.*, 2008). However, if tics are the main problem, then these should be treated first and foremost, which does not prevent us from evaluating comorbidities pre- and post-op by appropriate scales, as is done anyway in most current trials ( Kefalopoulou *et al.*, 2015). But we should be clear, for the time being, that obtaining a direct, surgically-induced effect on comorbidities will be the cherry on the cake, not something that can be systematically expected, at least based on our current knowledge of basal ganglia circuitry. That, for instance, was the rationale of the Paris group to implant electrodes into the limbic portions of the GPi, hoping to also reduce behavioral manifestations of GTS ( Houeto *et al.*, 2005; Welter *et al.*, 2008). In a similar vein, I am doubtful of implanting multiple electrodes in multiple sites in the hope of alleviating surgically a host of neuropsychiatric symptoms; although I admit that in rare, very debilitating cases, this might be an option to consider.

My take is rather this: having severe, relentless and debilitating tics tend to cloud comorbidities. In case of successful DBS, other symptoms, rather than being co-treated by electrode implantation, may actually re-emerge. However, the patient is now free to pursue other forms of treatment for these symptoms, for instance psychostimulants for the treatment of ADHD if these previously aggravated tics. Even more importantly, psychotherapeutic approaches thus far impossible, notably cognitive behavioural therapy (CBT), can become feasible. An example from the OCD world concerns patients who underwent a 24 week CBT treatment programme after DBS of the nucleus accumbens ( Mantione *et al.*, 2014). Not only did CBT offer further symptom improvement: rather, as the authors note, all patients (n=16) had undergone previous CBT trials (between 1 and 9) which were not only unsuccesful but sometimes counterproductive because they majored anxiety and fear. DBS appeared to alleviate these symptoms and thereby made successful CBT possible. In a similar vein, CBT aimed at further tic reduction could be tried post-op where, pre-op, it was unfeasible. The same applies for psychotherapeutical approaches aiming to improve OCD, depression, anxiety and behavioral problems.

Therefore, and in conclusion, I suggest to view DBS in GTS as a window or a stepping stone to a more holistic treatment rather than a single solution for all our patients’ ailments.
