# Supplementary material for: Deep brain stimulation in Gilles de la Tourette syndrome: killing several birds with one stone?
Source: F1000Res. 2016 Sep 7;5:2255. [Version 1] doi: 10.12688/f1000research.9521.1 (PMC5040147; doi:10.12688/f1000research.9521.1)
Supplement: Peer review history

# Peer review: Referee response for version 1

**DOI:** 10.5256/f1000research.10256.r16159

**Published:** 2016-09-28

**Authors:** Kevin J. Black, Shan Siddiqi

**Affiliations:** 1 Departments of Psychiatry, Neurology, Radiology, and Neuroscience, Washington University in St. Louis, St. Louis, MO, USA; 2 Department of Psychiatry, Washington University School of Medicine, St Louis, MO, USA

This is an interesting and thoughtful take on the approach to DBS in GTS. Because GTS is a complex neuropsychiatric syndrome with a variety of different symptoms (or comorbidities), it may not be reasonable to assume that all of these symptoms will be alleviated by DBS; to the contrary, they may actually appear to be worsening because they were previously being masked by the tics. As the author suggests, it is impossible to make accurate assessments of a treatment's effect without first appropriately classifying the phenomenology of the illness and/or symptoms being targeted.

 This article shows familiarity with the literature and clear writing, and is entirely appropriate for an Opinion Article.

 We add a few comments, hopefully to further the discussion. The author argues well for focusing clinical trials for DBS in GTS on one problem at a time, namely tics. However, as he notes, the great majority of patients with GTS have clinical features other than tics. Gilbert and Buncher (2005) ^1^ include this observation of multiple symptoms as one of several features that complicate performing and interpreting clinical trials in GTS. Focusing DBS on the most problematic symptom in each patient may even prove to produce better results than focusing DBS on tics. But we have insufficient data to make such a judgment. 

 The issues identified by Prof. Hartmann highlight the critical importance of further clinical trials and of registering all DBS experience in GTS ^2^.

 One trivial note: Substitute “exacerbated” for the word “majored” near the end of the article.

Shan H. Siddiqi

Kevin J. Black

We have read this submission. We believe that we have an appropriate level of expertise to confirm that it is of an acceptable scientific standard.

## References

[1] GilbertDLBuncherCR: Assessment of scientific and ethical issues in two randomized clinical trial designs for patients with Tourette's syndrome: a model for studies of multiple neuropsychiatric diagnoses. *J Neuropsychiatry Clin Neurosci*.2005;17(3) : 10.1176/jnp.17.3.324 324-32 10.1176/jnp.17.3.324 16179653

[2] DeebWRossiPJPortaMVisser-VandewalleVServelloDSilburnPCoyneTLeckmanJFFoltynieTHarizMJoyceEMZrinzoLKefalopoulouZWelterMLKarachiCMalletLHouetoJLShahed-JimenezJMengFGKlassenBTMogilnerAYPourfarMHKuhnJAckermansLKaidoTTemelYGrossREWalkerHCLozanoAMKhandharSMWalterBLWalterEMariZChangiziBKMoroEBaldermannJCHuysDZauberSESchrockLEZhangJGHuWFooteKDRizerKMinkJWWoodsDWGunduzAOkunMS: The International Deep Brain Stimulation Registry and Database for Gilles de la Tourette Syndrome: How Does It Work?. *Front Neurosci*.2016;10: 10.3389/fnins.2016.00170 170 10.3389/fnins.2016.00170 27199634PMC4842757

# Peer review: Referee response for version 1

**DOI:** 10.5256/f1000research.10256.r16161

**Published:** 2016-09-23

**Authors:** Jeremy Stern

**Affiliations:** 1 Department of Neurology, St George’s Hospital, St George's Hospital Medical School, London, UK

Professor Hartmann's opinion article is a clear-thinking and original critique of the directions that DBS for GTS is taking. The field is perhaps expanding faster than uncertainties are being addressed, partly due to the heterogenous nature of the condition and the possibility of several different surgical targets- variables that he surveys from an interesting perspective. I agree with him and Professor Cavanna that the practice of selecting modified surgical targets for tics based on comorbidites may lack a good evidence base, but the fundamental problem is establishing how to best to treat tics with DBS, including the prediction of beneficial effect for individual patients.The notion of DBS in GTS as an enabling therapy to allow conventional management strategies to be more successful needs further exploration and may prove an important principle.

I have read this submission. I believe that I have an appropriate level of expertise to confirm that it is of an acceptable scientific standard.

# Peer review: Referee response for version 1

**DOI:** 10.5256/f1000research.10256.r16164

**Published:** 2016-09-19

**Authors:** Andrea Cavanna

**Affiliations:** 1 Department of Neuropsychiatry, University of Birmingham, Birmingham, UK

I found Andreas Hartmann’s considerations on the use of Deep Brain Stimulation (DBS) for patients with Tourette syndrome (TS) both clinically sensible and thought-provoking. The article is clearly written and the take-home message is convincingly argued: patients with TS who are candidates to DBS present by definition with a clinical picture characterised by highly severe and refractory tics. Rather than considering DBS as a panacea for the multifaceted neurobehavioural spectrum complicating patients’ presentations, the focus (and expectations) of DBS should remain anchored to tic alleviation. From a practical point of view, it has been observed that the DBS procedure can have wide-ranging effects, however the approach of *a priori* targeting multiple symptoms at the same time (“killing several birds with one stone”) can be prone to theoretical and clinical fallacies. Conversely, it would be interesting to test the sequential approach proposed by the author by systematically assessing changes in health-related quality of life in patients undergoing tic-focused neuromodulation, followed by specific therapeutic interventions for the residual behavioural co-morbidities.

I have read this submission. I believe that I have an appropriate level of expertise to confirm that it is of an acceptable scientific standard.
